# A double blind randomized placebo control crossover trial on the effect of dietary nitrate supplementation on exercise tolerance in stable moderate chronic obstructive pulmonary disease

**DOI:** 10.1186/s12890-015-0057-4

**Published:** 2015-05-02

**Authors:** Paul Leong, Jane E Basham, Theresa Yong, Adrian Chazan, Paul Finlay, Sara Barnes, Phillip G Bardin, Donald Campbell

**Affiliations:** Monash Lung and Sleep, Monash Medical Centre, Clayton, VIC Australia; Department of General Medicine, Monash Medical Centre, 246 Clayton Road, Clayton, VIC 3168 Australia; Monash University, Clayton, VIC Australia

**Keywords:** COPD, Dietary nitrate, Exercise performance, Incremental shuttle walk test, Randomized control trial

## Abstract

**Background:**

Dietary nitrate supplementation has been shown to decrease the oxygen cost of exercise and prolong exercise tolerance, as measured by sub-maximal exercise endurance distance and time at 85% V̇O_2_max, in both elite athletes and normal healthy subjects. Patients with chronic obstructive pulmonary disease (COPD) have reduced quality of life and ability to perform activities of daily living attributable to diminished exercise tolerance, and dietary nitrate may be able to ameliorate this.

**Methods:**

We performed a double-blind, computer-randomized placebo control crossover trial at a tertiary Australian hospital to investigate whether dietary nitrate supplementation as beetroot juice (BR) would augment submaximal exercise endurance in individuals with spirometrically confirmed stable moderate COPD. Volunteers underwent an incremental shuttle walk test to determine V̇O_2_max followed by a test dose of BR to establish safety in the study population. Participants performed an endurance shuttle walk test (ESWT) at 85% V̇O_2_max after randomization to either a 3 day wash-in of BR (4.8 mmol twice a day) or placebo (nitrate deplete BR), with a final dose on the morning of testing. They then crossed over after 4 day washout. Repeated measures two sided paired t-tests were employed.

**Results:**

35 participants were recruited with 19 completing the trial. In the initial safety phase, we measured systolic blood pressure over four hours post first dose of BR, and found a mean 10 mmHg decrement maximal at 1 hour. One individual developed symptomatic postural hypotension and was excluded. The primary outcomes of ESWT distance and time to fatigue improved by 11% and 6% respectively; however these differences did not achieve statistical significance (p = 0.494 and 0.693 respectively).

**Conclusions:**

Our study does not support a role for routine dietary nitrate supplementation for enhancement of exercise endurance in COPD.

**Trial registration:**

Australia and New Zealand Clinical Trial Register: ACTRN12611001088932

**Electronic supplementary material:**

The online version of this article (doi:10.1186/s12890-015-0057-4) contains supplementary material, which is available to authorized users.

## Background

Chronic Obstructive Pulmonary Disease (COPD) is a leading cause of morbidity worldwide, with a substantial and increasing economic and social burden: it is estimated that 64 million people are affected worldwide [1,2]. Exercise intolerance and fatigue are major negative contributors to quality of life in individuals with COPD, for whom the activities of daily living may embody a significant exercise challenge [3].

Pharmacological inorganic nitrate (NO_3_^−^) supplementation reduces the O_2_ cost of and enhances high-intensity exercise tolerance in humans [4]. In a placebo-controlled study, dietary nitrate supplementation with beetroot juice (BR) reduced the O_2_ cost of submaximal walking and running exercise tolerance in young healthy males [5], and improves submaximal exercise endurance in club level cyclists [6]. A systematic review and meta-analysis of the effect of nitrate supplementation on exercise performance in healthy individuals has demonstrated that there is a significant moderate beneficial effect upon exercise performance as measured by time to exhaustion (effect size = 0.79 (95% CI, 0.23-1.35)). These benefits were more often observed in inactive to recreationally active individuals, and are present in both acute supplementation as well as chronic supplementation at up to at least 15 days [7].

Dietary nitrate supplementation appears to exert its effect via augmentation of the oxygen-independent entero-salivary pathway in which dietary NO_3_^−^ is reduced to biologically active nitrite (NO_2_^−^) and nitrous oxide (NO), resulting in greater NO availability [8]. This pathway is conceptualized as a ‘back-up’ to the oxygen-dependent classical L-arginine NO synthetase system, and is upregulated under acidemic or anaerobic conditions, situations conceivably more commonplace in individuals with COPD [9]. Resultant increased bioavailable NO may lead to enhanced vasodilation and/or O_2_ distribution within skeletal muscle, possibly by increasing the driving pressure of O_2_ in the microcirculation [10]. Other proposed mechanisms include increased muscle contractile efficiency and increased mitochondrial efficiency [11].

It has also been established that nitrate supplementation also lowers blood pressure, due to a vasodilatory effect [12,13]. This has safety implications for individuals with COPD who are often older, more frail and osteopenic and in whom a fracture may lead to life threatening complications [14]. The physiology of individuals with COPD greatly differs to healthy individuals, with a chronic inflammatory state resulting in disruption to pulmonary tissues, gas exchange abnormalities, cardiac dysfunction and skeletal muscle deconditioning [15]. Compared to healthy controls, individuals with COPD appear to have preserved baseline levels of nitrate and nitrite, but NO dynamics are altered, with differences in NO synthetase isoforms [16-18].

Three recent reports have reported the effects of acute and short term dietary nitrate supplementation on exercise endurance in individuals with COPD, with mixed results [19-21].

We performed a randomized, double blind placebo control crossover trial in order to test the hypothesis that short term dietary nitrate supplementation (3 days of 4.8 mmol twice daily) would exert a beneficial effect on exercise tolerance as measured by submaximal walk test endurance in individuals with moderate severity, stable COPD.

## Methods

Institutional ethics review was sought and received from the Southern Health Human Research Ethics Committee (11335A, approval date 24/1/2012). The trial was registered with the Australia and New Zealand Clinical Trial Registry (ACTRN12611001088932, registered 20/10/2011). All participants gave written informed consent prior to participation and refrained from antibacterial products including mouthwash and toothpaste during the study period. Apart from abstaining from beetroot products, no dietary restrictions were imposed. Participants’ medications remained unchanged for the duration of the trial.

BR was chosen as the source of dietary nitrate for this study for its ready commercial availability and use in prior literature. BEET-IT shots (James White Drinks, Ipswich, UK) were used for this study as the 0.3gm (4.8 mmol) dose of NO_3_^−^ in each 70 mL shot approximates the 0.34-0.38gm (5.5 mmol-6.2 mmol) NO_3_^−^ dose as described by prior investigators [4,5,22] without the requirement for a 500 mL daily fluid intake. Physically identical nitrate-deplete placebo shots (PL) were obtained from the manufacturer (0.00035-0.0013gm (0.0056-0.020 mmol) NO_3_^−^ dose) (personal communication, Pinho, A., James White Drinks, Ipswisch, UK). The dose administered was 70 mL (0.3gm NO_3_^−^), twice daily.

### Participants

Voluntary participants were recruited at a tertiary metropolitan Australian medical center either directly from the outpatient pulmonary function test laboratory or by direct referral from thoracic physicians. Inclusion criteria were Global Initiative for Chronic Obstructive Lung Disease (GOLD) class II stable COPD (‘moderate’ severity: FEV 1/FVC < 0.7, FEV 1 50-79% predicted) [1], age 45–80 and physical ability to perform an endurance shuttle walk test. GOLD II severity was chosen as a group who would be likely to experience exercise limitation but who would still be able to perform exercise testing. Exclusion criteria included a history of acute exacerbation of COPD in the preceding month, oral corticosteroid therapy, long term domiciliary O_2_ therapy, beta blocker therapy, ischemic heart disease or congestive cardiac failure and musculoskeletal problems limiting exercise.

### Baseline measurements

**Figure 1:**
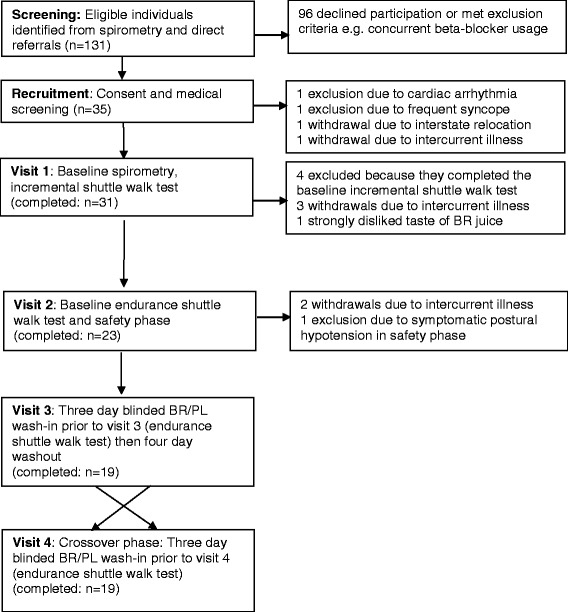
Study design. Visits 1 and 2 occurred approximately 1 week apart and collected baseline and safety data on participants. Visits 3 and 4 were exactly 7 days apart per study protocol. Prior to visit 3, participants consumed either beetroot (BR) or placebo (PL) twice a day for three days. They underwent an endurance shuttle walk test during visit 3 and then abstained from BR for 4 days to allow washout. In the crossover phase, participants were given to whichever juice (BR or PL) they had not previously been exposed to. This was taken twice a day for three days prior to visit 4, at which time another endurance shuttle walk test was performed.

At baseline (visit 1), participants underwent pre- and post-bronchodilator spirometry and measurement of carbon monoxide diffusing capacity as per American Thoracic Society/European Respiratory Society standard guidelines for pulmonary function testing [23] (Figure 1).

During the same visit, participants undertook an incremental shuttle walk test (ISWT) [24], a commonly used field walking test designed to simulate a maximal cardiopulmonary exercise test in individuals with COPD. This involved timed, accelerating shuttle walking around cones 9 m apart, forming a 10 m course. Standardized procedures were adopted as mandated by the ISWT protocol: these included uniform instructions and coaching and a repeat test to account for a learning effect. The best distance was recorded and used to estimate V̇O_2_max and to calculate the walking speed representing 85% of maximal exercise performance for use in the endurance shuttle walk test (ESWT) [25].

The ESWT [26] is a constant paced standardized test of endurance capacity developed for use in patients with COPD. It has face and construct validity as the exercise testing regime suitable validated for use in patients with COPD as a measure reflective of the workload undertaken by an individual with COPD in the course of their daily activities. Participants performed the walk under controlled conditions by walking at a constant predetermined rate around cones 9 m apart (10 m shuttle walk) to a constant, timed audio recording until exhaustion. The ESWT permits measurement of walking time and distance at the speed that represents their estimated 85% V̇O_2_max; hence it is a measure of the individual’s sub-maximal exercise endurance capacity.

Previous studies have demonstrated that it is not necessary to perform repeat determination of either the ISWT or the ESWT in this patient group [27].

### Initial ESWT and safety phase

During visit 2, which occurred approximately a week after visit 1, an initial ESWT was undertaken. Immediately after the ESWT, a Modified Borg Rating Scale for Perceived Dyspnea was recorded [28]. Following this, a safety phase was performed during which participants consumed, un-blinded, active BR and underwent measurement of sitting and standing blood pressure, heart rate and pulse oximetry saturations at 0, 0.5, 1 and 4 hours post dose. The rationale for this was that BR has been previously demonstrated to decrease resting systolic blood pressure [5]. As pre-specified, if participants became symptomatic or had a >20 mmHg systolic blood pressure fall in response to the test dose, they were excluded from the remainder of the study protocol.

### Double blinded randomized placebo controlled phase

Participants were randomized to receive either (active) BR or PL first in a double blind fashion by computerized allocation performed by an independent clinical trials pharmacist.

For three days prior to visit 3, participants consumed their allocated dose of juice (70 mL) twice per day. They consumed a final dose of juice on the morning of visit 3, approximately an hour prior to the performance of the ESWT. Per protocol, visits 3 and 4 occurred strictly 7 days apart.

Following visit 3, participants abstained from juice supplementation for four days, allowing a ‘wash-out’ period between dosing regimes. They then consumed whichever of BR or PL they had not previously been exposed to. This was again taken twice a day for three days prior to visit 4, with a final supplement on the morning of visit 4. Visit 4 also consisted of an ESWT.

The timing of supplementation was based on another randomized double-blind placebo-controlled crossover study [29], however washout was shortened from 10 to 7 days for logistical reasons.

Trial supplement adherence was checked by self-report and bottle count.

### Sample size estimation

A recent meta-analysis of the effect of nitrate supplementation on exercise performance in healthy individuals has demonstrated that there is a significant moderate beneficial effect upon exercise performance as measured by time to exhaustion with an effect size of 0.79 (95% CI, 0.23-1.35) [7]. Eaton et al. (2006) reported that the baseline performance by moderate severity COPD patients on the ESWT was 313 meters, with a standard deviation of 193 meters, and the reported improvement in performance in response to exercise rehabilitation of 92% of baseline (302 meters). However, using the reported 92% improvement in ESWT performance in response to pulmonary rehabilitation as the minimum clinically important difference (MCID) results in an effect size of 1.6, leading to a sample size requirement of only 6–7 patients in a cross-over study in order for the study to have an 80% power at the 5% significance level. A more conservative estimate of the MCID was used instead. We chose 55% improvement on the baseline performance in the ESWT as the MCID resulting in a more conservative estimate of the required sample size: 20 patients [30].

### Outcomes and statistical analysis

The pre-specified primary endpoints were distance walked (meters) and time to fatigue (minutes) on the ESWT on BR versus that on PL, with comparison by two-sided paired repeated sample t-tests. Secondary endpoints included safety phase data, Borg dyspnea scores and blood pressure. Analysis was performed using SPSS Statistics for Windows, Version 20.0 (Armonk, NY, IBM Corp, 2011). Results are presented as mean ± standard deviation, and statistical significance accepted if alpha <0.05.

## Results

### Recruitment

Between March 2012 and October 2013, 131 eligible participants were identified, 35 of whom agreed to participate in the study, 23 of whom completed the safety phase, and 19 went on to complete all four visits (Table 1 and Figure 1). One patient was excluded at enrolment as he was detected to have asymptomatic sick sinus syndrome. The most common reason for attrition during the study period was intercurrent illness, predominantly acute exacerbations of COPD.

**Table 1:** **Characteristics of participants who completed all four visits**

**N (completers)**	**19**
Sex	Female: 14 Male: 5
Age (years)	67 ± 7.9
Height (cm)	161.5 ± 8.1
Weight (kg)	76.5 ± 19.1
BMI	29.1 ± 6.5
FEV1 (% predicted)	62.0 ± 6.9
FVC (% predicted)	91.6 ± 12.9
FEV1/FVC	66.0 ± 7.6
TLCO (% predicted)	53.9 ± 13.9
V̇O_2_max (ml/min/kg)	14.9 ± 3.8

Abbreviations: FEV1: forced expiratory volume in 1 second, FVC: forced vital capacity, TLCO: transfer factor for carbon monoxide, V̇O_2_max: maximum oxygen uptake.

Four participants were assessed to be too fit for the trial following the initial ISWT. On the basis of completing the ISWT, these excluded participants’ predicted 85% V̇O_2_max exceeded the measurement ceiling of the ESWT (approximately 15 ml/min/kg). Any potential increase in walk distance therefore could not be measured with the ESWT. Therefore these four subjects were excluded. In the design phase, it was not foreseen that individuals with moderate COPD would be able to complete the ISWT.

### Baseline data

Of the 19 participants who completed the trial, the mean age was 67 and 14 were female. Their mean FEV1 was 62.0 ± 6.9% predicted, with TLCO of 53.9 ± 13.9% predicted. The mean estimated V̇O_2_max was 14.9 ± 3.8 ml/min/kg, with a calculated predicted 85% V̇O_2_max of 12.6 ± 3.2 ml/min/kg.

### Numbers analyzed and outcomes

The safety phase (n = 23) confirmed the known blood pressure effects of BR supplementation. Compared to sitting blood pressure at the start of the safety phase, there was a maximal mean systolic blood pressure decrement of 10 mmHg on standing at 1 hour (7.3%) (p = 0.001), accompanied by significant compensatory increases in heart rate at 0 hours (mean increase 6.2 ± 8.0 beats per minute, p = 0.001) and 0.5 hours (3.8 ± 7.5 beats per minute, p = 0.027) (Tables 2 and 3). One participant developed symptomatic postural hypotension at 4 hours (30 mmHg drop on sitting to standing at 4 hours) and was excluded from the trial. Diastolic blood pressure remained unchanged throughout the observation period (Additional file 1: Table S1).

**Table 2:** **Safety phase data for systolic blood pressure on standing (mmHg) (n = 23 unless otherwise stated)**

	**Systolic blood pressure**	**Mean difference from 0 hours sitting SBP**	Compared to 0 hours sitting SBP 95% CI*	**P value** ^**+**^
0 hours sitting	136.6 ± 17.6	n/a	n/a	n/a
0 hours standing	130.9 ± 16.7	−5.7 ± 9.5	−1.6 to −9.8	0.009^^^
0.5 hours standing	129.4 ± 13.4	−8.5 ± 14.7	−2.0 to −15.0	0.012^^^
1 hours standing^o^	127.6 ± 15.6	−10.0 ± 12.8	−4.5 to −16.0	0.001^^^
4 hours standing	129.0 ± 18.5	−7.5 ± 15.5	−0.8 to −14.3	0.029^^^

*95% confidence interval; ^+^paired sample two tailed *t*-test; ^^^significant comparison at the 95% confidence level; ^o^n = 22 for the 1 hour standing comparison due to missing data.

**Table 3:** **Safety phase data for heart rate (HR) on standing (beats per minute) (n = 23 unless otherwise stated)**

	**Heart rate**	**Mean difference from 0 hours sitting HR**	Compared to 0 hours sitting HR 95% CI*	**P value** ^**+**^
0 hours sitting	88.2 ± 11.9	n/a	n/a	n/a
0 hours standing^o^	94.3 ± 13.0	6.2 ± 8.0	2.8 to 9.7	0.001^^^
0.5 hours standing	92.4 ± 9.9	3.8 ± 7.5	0.47 to 7.1	0.027^^^
1 hours standing	92.0 ± 11.9	3.9 ± 10.0	−0.43 to 8.2	0.076
4 hours standing	87.7 ± 11.4	−0.478 ± 13.4	−6.3 to 5.3	0.866

*95% confidence interval; ^^^significant comparison at the 95% confidence level; ^+^paired sample two tailed *t*-test; ^o^n = 22 for the 0 hour standing comparison due to missing data.

19 participants completed the crossover paired sample phase of the trial (visits 3 and 4). Adherence to trial supplementation was excellent based on self report and bottle count, with the majority of participants reporting full compliance (n = 18) on both occasions and the remaining subject reporting only one missed dose during the three day wash-in period.

**Figure 2:**
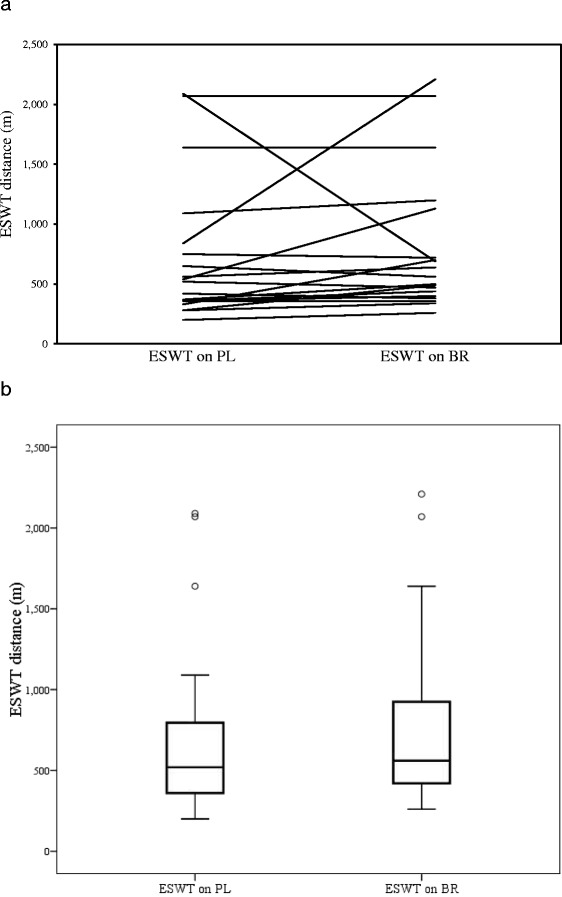
ESWT distance change. Figure 2**a**: Plot of individual participants’ endurance shuttle walk test (ESWT) distances (n = 19). Figure 2**b**: Box and stem plots of individual participants’ endurance shuttle walk test (ESWT) (n = 19). Abbreviations: BR: beetroot juice, PL: placebo.

Participants walked a mean distance of 721.6 ± 587.5 m on PL and 800.0 ± 584.3 m on BR (difference: 79 m, 11%) (Table 4). The median for PL was 520 m (interquartile range 360-795 m), and for BR, the median was 560 m (interquartile range 420-925 m) (Figure 2). These changes were not significant (p = 0.494, 95% CI −314.3 to 175.5 m). Similarly, walking time was not changed between PL (9.9 ± 7.5 minutes) and BR (10.5 ± 6.0 minutes) (difference = 0.6 minutes, 6%, p = 0.693). After walking, there was no difference in dyspnea score (Borg scale) or systolic blood pressure between the PL and BR treatment arms. These results did not change after removal of outliers (analysis not shown).

**Table 4:** **Study outcomes for pre-specified primary and secondary endpoints**

	**PL phase mean**	**BR phase mean**	95% CI*	**P value** ^**+**^
ESWT walking distance (m)	721.6 ± 587.5	800.0 ± 584.3	−314.3 to 157.5	0.494
ESWT walking time (min)	9.9 ± 7.5	10.5 ± 6.0	−3.4 to 2.3	0.693
Borg scale	3.41 ± 2.0	3.1 ± 1.6	−0.2 to 0.9	0.244
Systolic blood pressure prior to ESWT (mmHg)	132 ± 16.2	134.6 ± 18.2	−12.9 to 7.8	0.962
Systolic blood pressure after ESWT (mmHg)	159.8 ± 26.6	169.1 ± 29.7	−29.1 to 10.5	0.336

*95% confidence interval; ^+^paired sample two tailed *t*-test.

## Discussion

The results of this study indicate that dietary nitrate supplementation in individuals with moderate COPD a) does not significantly increase submaximal exercise endurance as measured by ESWT distance, time or dyspnea score and b) decreases resting systolic blood pressure with c) compensatory increases in resting heart rate.

### Limitations

Unfortunately given logistical (funding) constraints, it was impractical to measure serum NO3^−^ and NO2^−^ levels: ideally, these would have been measured at baseline and post supplementation, with additional analyses carried out to determine whether improvements in exercise endurance were associated with increased NO3^−^ and/or NO2^−^, and whether medications or comorbidities interacted with this effect. We observed a decrement in blood pressure in the active arm, suggestive of successful nitrate supplementation, however, quantitative determination of NO3^−^ and/or NO2^−^ may have provided definite confirmation. Confirmation of return of NO3^−^ and NO2^−^ to baseline values after the four day washout period may also have been useful.

Our trial also suffered from a high attrition rate: from 35 subjects, only 19 completed the trial. This is significantly higher than in other trials of dietary nitrate supplementation in individuals with COPD, in which withdrawals were limited to a maximum of 1 per trial [19-21]. The attrition rate in our study is reflective of the burden of illness in individuals with COPD, with multiple drop-outs due to underlying illness or intercurrent illness. 10 of our patients withdrew due to medical illness (predominantly COPD exacerbations), and in the safety phase, one withdrew with symptomatic postural hypotension induced by BR, confirming the reported [5,12] effect of dietary nitrates on blood pressure. These withdrawals may reflect the tertiary nature of our center, with a high proportion of more unwell patients.

### Comparisons

The dose and timing of nitrate used in our study differed to that used by other investigators who have examined its effect on exercise endurance in COPD. Kerley et al. and Berry et al., both of whom found positive effects, used acute doses of 12.9 mmol and 7.58 mmol respectively [19,20]. However, Shepherd et al. used 6.77 mmol twice a day for 2.5 days and found no difference [21]. We used 4.8 mmol twice a day for three days with a further dose on the morning of walk test and found no significant difference. Although a trial in healthy cyclists used a wash-in period and demonstrated benefit (three days 0.1 mmol nitrate/kg/day) [29], it is possible that physiological alterations in aging, or in COPD render wash-in periods less useful, or alternatively, that the dose we used was too low.

The effect of increased adiposity has been proposed as a modifying factor in the response to dietary nitrate, by means that are unknown [21]. The BMI (kg/m^2^) of our study population (29.1 ± 6.5) was similar to that in Berry et al. (29.2 ± 5.5) who found similar an increase in exercise performance. Kerley et al. had a slightly lighter population and found an increase in exercise endurance with a BMI of 27.3 ± 6.4, whereas Shepherd et al., whose patients were heavier than ours, and found no difference with a BMI of 30.8 ± 3.2. The influence of adiposity on nitrate supplementation’s effect on exercise supplementation remains yet to be defined.

In healthy older individuals, short term dietary nitrate supplementation at 9.6 mmol/day reduced resting blood pressure but there was no effect on walk time, an effect broadly similar to our findings [31]. Interestingly, although decreases in the oxygen cost of submaximal exercise have been relatively consistently found in younger individuals [7], the oxygen cost of submaximal exercise was not decreased in those healthy older individuals, for reasons that are unclear. Additionally, decreases in the oxygen cost of exercise were not found in the studies of nitrate supplementation of individuals with COPD, suggesting that if exercise endurance is increased in COPD, the mechanisms may be different to that in younger individuals [20,21].

We found a heterogeneous response to dietary nitrate in our study population, consistent with Berry et al. who, despite an overall positive effect, found that two of their 11 participants had a decrease in exercise time. This variation in response was also noted by Kerley et al., and its mechanism remains unclear. It may therefore be that individuals with COPD differ in their response to dietary nitrate and that our study population may have been comprised of a greater proportion of non-responders.

Skeletal muscle in individuals with COPD may affected by myriad factors, which include deconditioning, hypoxia, hypercapnia, systemic inflammation, malnutrition, and drug therapy. Multiple abnormalities result, including redox imbalance, autophagy induction, mitochondrial dysfunction, a protein catabolic state with reduced anabolism and structural abnormalities [32]. Intriguingly, it also appears that epigenetic modification may play a role in muscle phenotype and performance in COPD [33]. Additionally, exercise limitation may result from not only skeletal muscle factors, but also lung and haemodynamic factors [34]. These complex, interwoven factors and their varying extents in any given individual with COPD may make prediction of response to dietary nitrate supplementation difficult, and furthermore may render it challenging to precisely identify a mechanism for exercise enhancement, if it exists.

### Study design

An important question arising from this study is whether the observed difference in endurance distance (11%) and time to fatigue (6%) is clinically important. A change in distance of 60-115 m has been reported as a minimally clinically important difference in the ESWT in the context of a pharmacological trial [35]. In our study, the observed incremental improvement in ESWT was only 79 m whilst the standard deviation of the observed values for in the baseline ESWT was 583 m. These values represent a much reduced effect size of approximately 14% compared with our initial estimate of 55%. Were the sample size larger and the standard deviation reduced, the change in distance may have been clinically significant. An important question is therefore whether the study was under-powered.

Study sample size estimates prior to this study were based on estimates of effect sizes derived from the published evidence. A meta-analysis of the effect of nitrate supplementation on exercise performance in healthy individuals has demonstrated that there is a significant moderate beneficial effect upon exercise performance as measured by time to exhaustion (effect size = 0.79 (95% CI, 0.23-1.35) [7]). Estimates of the effect size derived from studies of the incremental effect of pulmonary rehabilitation program upon exercise distance and time in the endurance shuttle walk test ESWT yielded an effect size of 1.6 [36]. This was deemed to be a clinically important incremental improvement, and was therefore used as a guide for the choice of effect size for the power calculation for this study. We used the published ESWT data to inform us of the anticipated incremental improvement and the standard deviation for baseline performance.

The sample size we chose reflected favorably with the sample sizes chosen for recently reported studies of dietary nitrate supplementation in COPD, in which sample size ranged from 11–15 [19-21]. Our trial, with a final sample size of 19, did not demonstrate an effect of dietary nitrate supplementation in COPD, concordant with the results of Shepard et al. (n = 13) [21]. This contrasts with the results of Berry et al. and Kerley et al., who found a positive effect on exercise performance for dietary nitrate supplementation in COPD, and whose sample sizes were 15 and 11 respectively [20,31].

## Conclusion

Our results do not support a role for the routine use of acute dietary nitrate supplementation in individuals with GOLD stage II COPD for the purposes of physical activity limitation. Whilst this trial, to our knowledge, is the largest trial of nitrate supplementation in individuals with COPD, other investigators have reported positive effects on exercise endurance for dietary nitrate supplementation in COPD. A consistent finding is decreased blood pressure, and with the development of symptomatic postural hypotension seen in one individual in our study, the role and risk-benefit ratio of acute dietary nitrate supplementation remains undefined. Overall, it is difficult draw a firm conclusion as to whether acute dietary supplementation for exercise endurance in COPD is effective given study heterogeneity (nitrate dose and form, study setting, exercise methodology).

### Future directions

Given the discordance between this study and other recently published literature, further studies should recruit larger patient cohorts, in order to minimize the effect of type II error and to examine an interaction between nitrate, medications and comorbidities. The identification of a dose–response relationship for both exercise endurance and hypotension would be important if an effect for the former were to be confirmed and nitrate were to be extended to routine clinical use. Further pharmacodynamics studies could also establish whether tolerance develops. An important step would be to identify whether there are distinct phenotypes or subpopulations of COPD that can be classified into nitrate responders or non-responders, and in whom hypotension may be an issue.

An important study to perform in the future may be to examine for a potential interaction in effects on submaximal exercise endurance between a pulmonary rehabilitation program and dietary nitrate supplementation. Such a study will likely need to be a multicenter randomized double blind placebo controlled study enrolling large numbers of subjects to be recruited given the likely drop out rate may approach 50%. The results of this study may help inform sample size estimates for such a study.

## Additional file

Additional file 1: Table S1. Safety phase data for diastolic blood pressure (DBP) on standing (mmHg) (n=23 unless otherwise stated).

## Abbreviations

BR: Beetroot juice
COPD: Chronic obstructive pulmonary disease
ESWT: Endurance shuttle walk test
FEV1: Forced expiratory volume in 1 second
FVC: Forced vital capacity
ISWT: Incremental shuttle walk test
NO: Nitrous oxide
NO_2_^−^: Nitrite
NO_3_^−^: Nitrate
PL: Placebo
TLCO: Transfer factor for carbon monoxide
V̇O2max: Maximum oxygen uptake
