# Supplementary material for: A double blind randomized placebo control crossover trial on the effect of dietary nitrate supplementation on exercise tolerance in stable moderate chronic obstructive pulmonary disease
Source: BMC Pulm Med. 2015 May 2;15:52. doi: 10.1186/s12890-015-0057-4 (PMC4423518; doi:10.1186/s12890-015-0057-4)
Supplement: Additional file 1: Table S1. — Safety phase data for diastolic blood pressure (DBP) on standing (mmHg) (n=23 unless otherwise stated). [file 12890_2015_57_MOESM1_ESM.docx]

**For additional file**

**Additional Table 1: Safety phase data for diastolic blood pressure (DBP) on standing (mmHg) (n=23 unless otherwise stated)**

|  | Diastolic blood pressure | Mean difference from 0 hours sitting DBP | Compared to 0 hours sitting DBP  95% CI* | P value^+^ |
| --- | --- | --- | --- | --- |
| 0 hours sitting | 76.6±14.3 | n/a | n/a | n/a |
| 0 hours standing | 79.5±11.2 | 3.0±12.3 | -2.4 to 8.3 | 0.263 |
| 0.5 hours standing^o^ | 78.7±9.4 | 1.6±12.7 | -4.1 to 7.2 | 0.565 |
| 1 hours standing | 76.7±10.0 | 0.1±14.6 | -6.2 to 6.4 | 0.966 |
| 4 hours standing | 79.2±10.3 | 2.7±13.4 | -3.1 to 8.4 | 0.352 |

*95% confidence interval; ^+^paired samples two tailed t-test; ^o^ n=22 for the 0.5 hour standing comparison due to missing data
